# Galectin-1-mediated high NCAPG expression correlates with poor prognosis in gastric cancer

**DOI:** 10.18632/aging.204806

**Published:** 2023-06-16

**Authors:** Tingrui Zheng, Tao Qian, Haihua Zhou, Zhiyi Cheng, Guiyuan Liu, Chuanjiang Huang, Rongrong Dou, Fuxing Liu, Xiaolan You

**Affiliations:** 1 Department of Gastrointestinal Surgery, The Affiliated Taizhou People’s Hospital of Nanjing Medical University, Taizhou 225300, Jiangsu, China; 2 Department of Anesthesiology, The Affiliated Taizhou People’s Hospital of Nanjing Medical University, Taizhou 225300, Jiangsu, China; 3 Department of the Pathology, The Affiliated Taizhou People’s Hospital of Nanjing Medical University, Taizhou 225300, Jiangsu, China

**Keywords:** gastric cancer, Galectin-1, NCAPG, prognosis, metastasis

## Abstract

Galectin-1 (Gal1) and non-SMC condensin I complex, subunit G (NCAPG) are associated with metastasis in several malignant tumors. However, their precise roles in gastric cancer (GC) remain uncertain. This study explored the clinical significance and relationship of Gal1 and NCAPG in GC. Gal1 and NCAPG expressions were significantly up-regulated in GC compared to adjacent non-cancerous tissues by immunohistochemistry (IHC) and Western blotting. Besides, methods including stable transfection, quantitative real-time reverse transcription PCR, Western blotting, Matrigel invasion and wound-healing assays *in vitro*, were also conducted. IHC scores for Gal1 and NCAPG had a positive correlation in GC tissues. High Gal1 or NCAPG expression significantly correlated with poor prognosis in GC, and Gal1 combined with NCAPG had a synergetic effect on the prediction of GC prognosis. Gal1 overexpression *in vitro* enhanced NCAPG expression, cell migration, and invasion in SGC-7901 and HGC-27 cells. Simultaneous Gal1 overexpression and NCAPG knockdown in GC cells partly rescued the migrative and invasive abilities. Thus, Gal1 promoted GC invasion through increased NCAPG expression. The present study demonstrated the prognostic significance of the combination of Gal1 and NCAPG in GC for the first time.

## INTRODUCTION

Gastric cancer (GC) has high morbidity and mortality rates worldwide [1]. The incidence of GC varies greatly in different parts of the world, with high incidence in Asian countries, including China, Japan, and South Korea [1, 2]. More than one million new GC cases are reported annually, 40% of which occur in China. Most of the GC-related deaths also occur in China, which poses a serious threat to the health of Chinese people [1].

The comprehensive treatment for GC includes surgery, chemotherapy, radiotherapy, molecular targeted therapy, biological immunotherapy, and traditional Chinese medicine [3]. Despite the emergence of newer treatments, the recurrence and metastasis rates for advanced GC remain high, which leads to mortality in GC patients. Recurrence and metastasis in GC are complex, multi-step processes regulated by several genes, including oncogenes and tumor suppressor genes [4–6]. An understanding of the regulatory genes and mechanisms of GC recurrence and metastasis may facilitate effective treatment.

Galectin-1 (Gal1) is one of the 15 members of the beta-galactose-binding proteins, galactoagglutinins. Recent studies have demonstrated that Gal1 is expressed in a variety of malignant tumors, including hepatocellular carcinomas [7], lung adenocarcinomas [8], pancreatic cancers [9], breast cancers [10], colon cancers [11], and GC [12]. It is mainly distributed in the extracellular matrix and cytoplasm, and regulates various biological activities of cancer cells. It promotes the occurrence, development, invasion, metastasis, angiogenesis, immune escape, and other biological functions of malignant tumors [13]. However, the mechanism by which Gal1 regulates the biological behavior of tumors is not completely understood.

Non-SMC condensin I complex, subunit G (NCAPG) is a mitotic gene located on human chromosome 4p15.32 and has a relative molecular weight of 114.1 kDa. Studies have shown that NCAPG expression varies among tissues, with high expression in testicular tissues and low expression in thymus. Its expression is also detectable in various tumors [14–17]. NCAPG overexpression is related to the proliferation and migration of hepatocellular carcinomas [18]. NCAPG expression is significantly higher in GC compared to the adjacent tissues, and it influences the prognosis of GC patients [19]. However, the association between NCAPG and Gal1 in GC patients has not been reported.

The present study focused on the role of Gal1 and NCAPG in GC and their effects on invasion and metastasis. We demonstrated that Gal1 and NCAPG could be used as predictors of GC prognosis. Their combination as a novel predictor had a high accuracy for survival assessment.

## MATERIALS AND METHODS

### Patient information and tissue samples

A total of 145 gastric adenocarcinoma patients, with detailed pathological and follow-up data were enrolled in this study. All patients were treated with radical gastrectomy and D2 lymphadenectomy at the Gastrointestinal Surgery Department, Taizhou People’s Hospital of Jiangsu province between January 2015 and May 2017. No patient received radiotherapy or chemotherapy prior to surgery, had no serious diseases or other synchronous malignancies, or distant metastases prior to surgery. Overall survival (OS) and disease free survival (DFS) times were the primary endpoints. OS was calculated from the date of surgery to the date of death or the final follow-up. DFS was calculated from the date of surgery to disease recurrence. The clinicopathological features of the patients are described in Table 1. Primary GC tissues (GCTs) and matched normal gastric mucosa tissues (NGCTs) were formalin-fixed and paraffin-embedded for hematoxylin and eosin (HE) staining and immunohistochemistry (IHC), while fresh GCTs and NGCTs were collected from eight patients for molecular analysis in April 2022.

**Table 1:** Relationship between expression levels of Gal1 or NCAPG and clinicopathological features in patients with GC.

**All patients**	**Gal1**			**NCAPG**		
	**High (%)**	**Low (%)**	***P***	**High (%)**	**Low (%)**	***P***
	***n* = 83**	***n* = 62**		***n* = 110**	***n* = 35**	
Sex			0.142			0.019
Male	52 (62.7)	46 (74.2)		80 (72.7)	18 (51.4)	
Female	31 (37.3)	16 (25.8)		30 (27.3)	17 (48.6)	
Age (years)			0.765			
>65	45 (54.2)	32 (51.6)		59 (53.6)	18 (51.4)	0.82
≤65	38 (45.8)	30 (48.4)		51 (46.4)	17 (48.6)	
Tumor diameter (cm)			0.004			0.027
>5	35 (42.2)	12 (19.4)		41 (37.3)	6 (17.1)	
≤5	48 (57.8)	50 (80.6)		69 (62.7)	29 (82.9)	
Pathological classification			<0.001			
I–II	7 (8.4)	31 (50.0)		16 (14.5)	22 (62.9)	0.001
III	76 (91.6)	31 (50.0)		94 (85.5)	13 (37.1)	
Depth of invasion			0.01			
T2–T3	9 (10.8)	17 (27.4)		14 (12.7)	12 (34.3)	0.004
T4	74 (89.2)	45 (72.6)		96 (87.3)	23 (65.7)	
Lymph node metastasis			0.001			0.037
N0	21 (25.3)	34 (54.8)		35 (31.8)	20 (57.2)	
N1	15 (18.1)	9 (14.5)		18 (16.4)	6 (17.1)	
N2	17 (20.5)	11 (17.8)		24 (21.8)	4 (11.4)	
N3	30 (36.1)	8 (12.9)		33 (30.0)	5 (14.3)	
TNM stage			0.011			
I	4 (4.8)	13 (21.0)		9 (8.2)	8 (22.9)	0.015
II	5 (6.0)	4 (6.4)		5 (4.5)	4 (11.4)	
III	74 (89.2)	45 (72.6)		96 (87.3)	23 (65.7)	

### IHC staining

Formalin-fixed, paraffin-embedded specimens were used for IHC analysis. The speciments were sliced at 4-μm thickness. Xylene and gradient ethanol were used to deparaffinize and rehydrate the tissues. Endogenous peroxidases were blocked in methanol for 10 min using 3% hydrogen peroxide. Phosphate-buffered saline (PBS) was used to wash the slides three times, and citrate buffer (pH 6.0) was used for antigen retrieval for 20 min at 95°C. Rabbit monoclonal antibodies, anti-Gal1 (dilution, 1:250, Abcam, Cambridge, UK) and NCAPG (dilution, 1:200, Abcam), were incubated with the slides overnight at 4°C. The slides were washed thrice with PBS and incubated with biotin-conjugated secondary antibodies, followed by horseradish peroxidase-conjugated streptavidin. The sections were then stained with diaminobenzidine, counterstained with hematoxylin, dehydrated, cleared, and cover-slipped.

### IHC analysis

Two pathologists, who were blinded to the clinical data, scored the IHC staining for Gal1 and NCAPG in GCTs and NGCTs using semi-quantitative immunoreactivity scores (IRS). Immunostaining intensity was documented as category A with scores of 0–3 (0, negative; 1, weak; 2, moderate; and 3, strong). The percentage of immunoreactive cells was documented as category B with scores of 1–4 (1, 0–25%; 2, 26–50%; 3, 51–75%; and 4, 76–100%). IRS was calculated by multiplying the scores for categories A and B, ranging from 0 to 12. Receiver operating characteristic (ROC) analysis was used to obtain the optimum cutoff values for IRS and to distinguish high and low expression of Gal1 and NCAPG.

### Cell lines and culture

Human GC cells, SGC-7901 (Cat No. C6795), and undifferentiated GC cells, HGC-27 (Cat No. C6365), were provided by Shanghai Biyuntian Biological Co., Ltd. (Shanghai, China). RPMI-1640 (Gibco-BRL, Gaithersburgh, MD, USA) with 10% (v/v) fetal bovine serum (Gibco-BRL), 100 mg/mL of streptomycin, and 100 U/mL of penicillin (Gibco-BRL) were used to culture GC cells. All cells were cultured in a humidified atmosphere containing 5% (v/v) CO_2_ at 37°C, and passaged by trypsinization when 80% confluence was reached.

### Lentiviral transduction

A lentiviral vector for *LGALS1* overexpression and a corresponding non-targeting negative control lentiviral vector were constructed by Genechem Co., Ltd. (Shanghai, China). The GV358 (Ubi-MCS-3FLAG-SV40-EGFP-IRES-puromycin) lentiviral vector was constructed to upregulate *LGALS1* expression. SGC-7901 and HGC-27 cells were seeded at a concentration of 5 × 10^4^ cells per well in six-well plates before lentiviral transduction. The cells were transduced with lentiviral vector and 10 μg/mL of polybrene (Sigma-Aldrich, St. Louis, MO, USA) based on an infection multiplicity of 10. The corresponding non-targeting negative control lentiviral vector was transduced through the same approach. The medium was replaced 12 h after transduction and puromycin (Sigma-Aldrich) was added to select stable transduced cell lines at the concentration of 2 μg/mL after another 48 h. The stable transduced cells were then cultured in the presence of 0.5 μg/mL of puromycin. Fluorescent microscopy (OLYMPUS-U-HGLGPS-IX73), qualitative reverse-transcriptase polymerase chain reaction (qRT-PCR), and Western blotting were used to evaluate transduction efficiency after 72 h.

### siRNA transduction

The siRNA against *NCAPG* and matched negative control siRNA were purchased from Biomics Biotechnologies Co. Ltd. (Nantong, China). OE-*LGALS1* SGC-7901 and OE-*LGALS1* HGC-27 cells were seeded in six-well plates at a concentration of 5 × 10^4^, and were transfected with *NCAPG* siRNA or control siRNA using Lipofectamine 2000 (Invitrogen, Carlsbad, CA, USA). The manufacturer’s instructions were followed for all the steps. After 24 h, the cells were harvested for further experiments. The three siRNA sequences were as follows: hs-*NCAPG*-si-1 sense (5′–3′): CACGAUGGAUGAUAAGACA, hs-NCAPG-si-1 antisense (3′–5′): UGUCUUAUCAUCCAUCGUG; hs-NCAPG-si-2 sense (5′–3′): GGAGUUCAUUCAUUACCUU, hs-NCAPG-si-2 antisense (3′-5′): AAGGUAAUGAAUGAACUCC; hs-NCAPG-si-3 sense (5′–3′): GCUGAAACAUUGCAGAAAU, and hs-NCAPG-si-3 antisense (3′–5′): AUUUCUGCAAUGUUUCAGC.

### Wound healing assay

All cells were seeded in six-well plates at concentrations of 1 × 10^5^. When the cell monolayer reached a confluence of 80–90%, a 10-μL pipette tip was scored across it to create a wound. PBS (Corning, Manassas, VA, USA) was used to wash the plates and remove cellular debris. The cells were photographed using a Leica DMIRB microscope (100× magnification; Leica, Wetzlar, Germany). Then, the cells were incubated with a serum-free medium containing 10 μg/mL of mitomycin C to block proliferation, and the wounds were photographed after 24 h. The number of migrated wild type cells was defined as 100% to calculate the percentage of cell migration.

### *In vitro* invasion assay

The invasion bility of SGC-7901 and HGC-27 cells was measured using 24-well transwell units with polycarbonate filters (pore size, 8.0 μm; Corning, NY, USA). Matrigel^®^ basement membrane (BD Biosciences, San Diego, CA, USA) was mixed with the serum-free RPMI at a ratio of 1:8, and 100 μL of the mixture was used to coat the upper transwell inserts. Then, the cells were seeded at 1 × 10^5^ in the upper insert with 100 μL serum-free RPMI medium, and placed in the lower chambers with 600 μL of complete media. The cells were allowed to culture for 24 h at 37°C, and non-invasive cells were removed using a cotton swab. The filters were fixed using 4% (v/v) paraformaldehyde, the cells were stained with 0.05% (v/v) crystal violet solution, and counted under a microscope.

### RNA extraction and real-time PCR

The RNeasy Mini Kit (Invitrogen) was used to isolate real-time PCR RNAs, and the Revert Aid RT reverse transcription kit (Thermo Fisher Scientific, Waltham, MA, USA) was used to reverse-translate the purified RNAs into cDNA. A SYBR Green dye kit (RocheDiagnostics, Mannheim, Germany) was used to perform qRT-PCR, and the products were analyzed using an iQ5 Multicolor real-time PCR Detection System (Bio-Rad, Hercules, CA, USA). GAPDH was used as the reference control gene and analyzed using the 2^−ΔΔCt^ method. The following primers were used: *NCAPG* (forward): ACCCAAGCATCAAAGTCTACTCAGC and (reverse) TGACACCTCCTGTTCGTCCTAGC; *LGALS1* (forward): GCCAGATGGATACGAATTCAAG and GCCACACATTTGATCTTGAAGT; and *GAPDH* (forward) CCAGCAAGAGCACAAGAGGAAGAG and (reverse) GGTCTACATGGCAACTGTGAGGAG.

### Western blotting

The tissues and cells were lysed using the RIPA buffer (Thermo Fisher Scientific) to prepare total cells extracts. Denatured proteins were separated by 10% sodium dodecyl sulfate-polyacrylamide gel electrophoresis (SDS-PAGE) and the separated proteins were transferred onto nitrocellulose membranes (GE Healthcare Life Sciences, Pittsburgh, PA, USA). Antibodies against Gal1, NCAPG, and GAPDH (dilution, 1:2000) were used to probe the proteins on the blots at 4°C overnight. They were then incubated with peroxidase-conjugated secondary anti-bodies (Sigma-Aldrich), and a West Pico chemiluminescent substrate (Pierce, Carlsbad, CA, USA) was used to visualize the immunoreactive protein bands. A densitometric image analysis software (Image Master VDS; Pharmacia Biotech, Little Chalfont, UK) was used to quantify the proteins. GAPDH levels were determined as an internal reference. All experiments were independently performed thrice.

### Statistical analyses

SPSS Statistics version 25.0 software (IBM Corp., Armonk, NY, USA) was used to analyze the data. The clinicopathological features and protein expression levels were compared using the chi-square test. Wilcoxon test (grouped) was used to analyze the IRS for Gal1 and NCAPG in GCTs and NGCTs. OS and DFS were analyzed through Kaplan-Meier survival analysis. The hazard ratios (HRs) and 95% confidence intervals (CIs) were estimated by univariate or multivariate Cox regression analysis. Continuous variables were expressed as means ± standard error of the mean. Multiple comparisons were performed through one-way analysis of variance (ANOVA) and Dunnett’s *t* test. *P*-values < 0.05 were considered significant.

## RESULTS

### Gal1 and NCAPG expression in GC and non-cancer tissues

**Figure 1:**
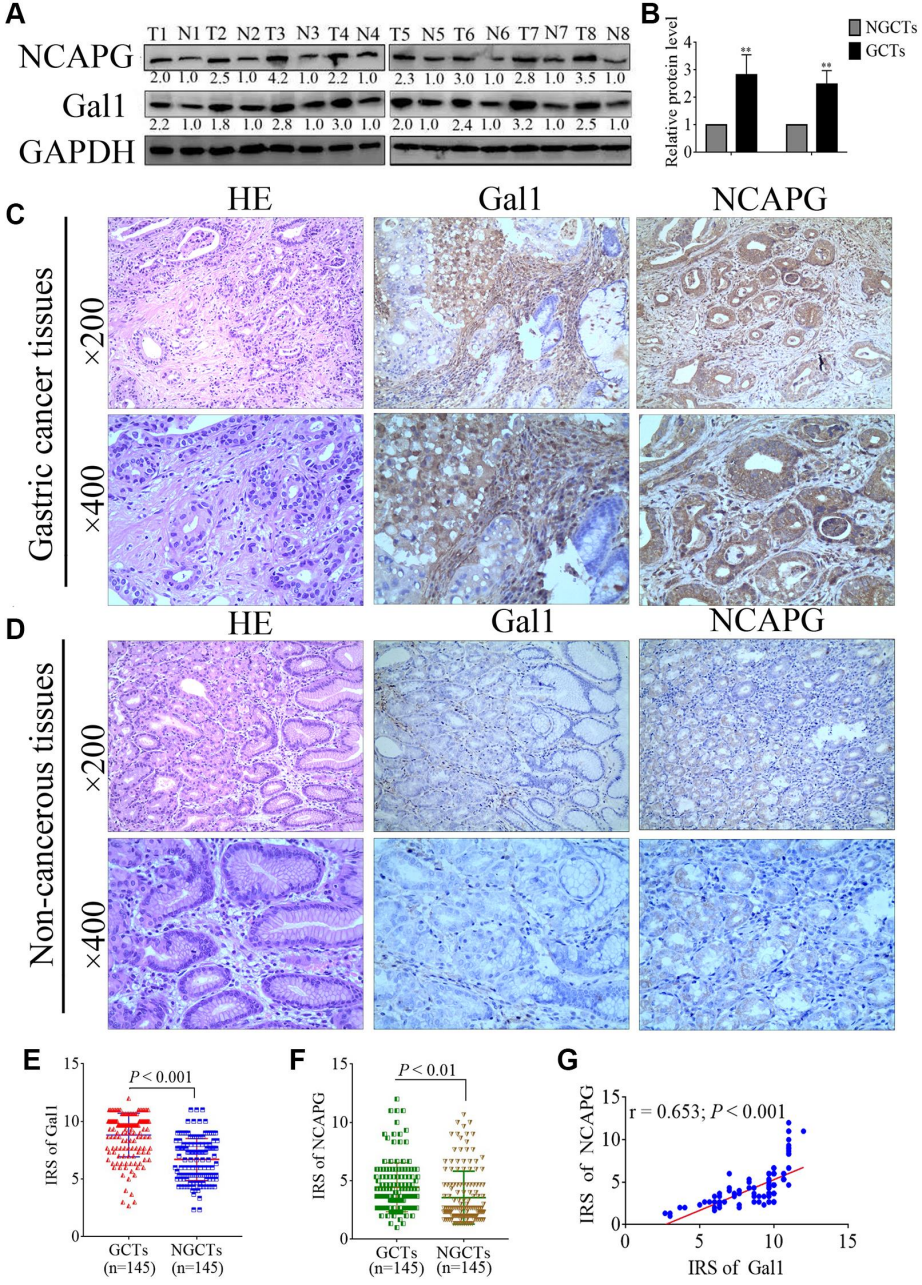
**Gal1 and NCAPG expression predict prognosis of gastric cancer (GC).** (**A**, **B**) Expression of Gal1 and NCAPG proteins detected by Western blotting in GC cancer tissues (GCTs) and non-gastric cancer tissues (NGCTs). (**C**, **D**) Representative immunohistochemistry images for Gal1 and NCAPG in (**C**) GC tissues, and (**D**) matched non-cancerous tissues. (**E**) Immunoreactivity score (IRS) for Gal1 compared between GCTs and matched NGCTs. (**F**) IRS for NCAPG compared between GCTs and matched NGCTs. (**G**) The expression of Gal1 was positively correlated with NCAPG expression in GC tissues. Abbreviations: T: tumor tissue; N: non-tumor tissue; GCTs: gastric cancer tissues; NGCTs: non- gastric cancer tissues. ^**^P < 0.01.

Western blotting was used to analyze Gal1 and NCAPG expressions in eight paired specimens from GC patients, including primary GCTs and matched NGCTs. GCTs had increased Gal1 and NCAPG expressions compared to the paired NGCTs (Figure 1A, 1B). Immunohistochemistry of the GCTs and NGCTs was used to further investigate Gal1 and NCAPG expressions in 145 primary GC patients. Gal1 staining was mainly localized in the cytoplasm and extracellular matrix, whereas NCAPG was mainly expressed in the cytoplasm and nuclei (Figure 1C). Typical images of Gal1 and NCAPG expression in non-cancer tissues are shown in Figure 1D. The IRS distribution for Gal1 and NCAPG expressions in primary GCTs and NGCTs are shown in Figure 1E, 1F. Compared to NGCTs, Gal1 and NCAPG expressions in GCTs were significantly increased (both *P* < 0.001, Figure 1E, 1F). NCAPG expression correlated significantly with Gal1 expression in cancerous tissues (r = 0.653, *P* < 0.001, Figure 1G).

### Gal1 and NCAPG expression correlated with clinicopathological characteristics

**Figure 2:**
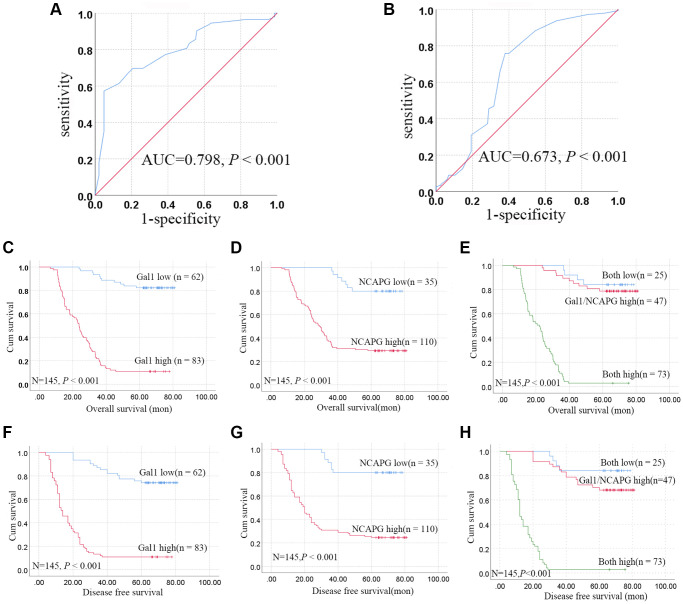
**Aberrant Gal1 and NCAPG expression in GC tissues indicates poor prognosis.** (**A**, **B**) Receiver operating characteristic statistics were employed to estimate the cut-off points of the IRS for (**A**) Gal1 and (**B**) NCAPG in GC tissues. (**C**–**E**) Kaplan-Meier curves for Gal1, NCAPG, and combined Gal1/NCAPG expression in the training cohort for overall survival. (**F**–**H**) Kaplan-Meier curves for Gal1, NCAPG, and combined Gal1/NCAPG expression in the training cohort for disease free survival.

IHC cutoff scores were used to classify Gal1 and NCAPG levels as high and low through ROC analysis. IRS ≥ 9.17 and ≥ 3.17 indicated high Gal1 (Figure 2A) and NCAPG (Figure 2B) expressions, respectively. Based on this standard, high Gal1 and NCAPG expression was 83 (57.24%) and 110 (75.86%) in GC cases, respectively. Gal1 expression in GCTs correlated with the clinicopathological features (Table 1), including tumor diameter, pathological classification, depth of invasion, lymph node metastasis, and TNM stage (all *P* < 0.05). NCAPG expression in GCTs was significantly associated with gender, tumor diameter, pathological classification, depth of invasion, lymph node metastasis, and TNM stage (all *P* < 0.05).

### High Gal1 and NCAPG expression inversely correlated with survival

All patient follow-up ended at death or at least 5 years, the median follow-up time was 36.37 months. Kaplan-Meier analysis revealed that high Gal1 or NCAPG expression in GCTs was significantly correlated with poor OS (all *P* < 0.001, Figure 2C, 2D) and DFS (all *P* < 0.001, Figure 2F, 2G). Univariate Cox regression analysis indicated that tumor diameter, pathological classification, depth of invasion, lymph node metastasis, TNM stage, and Gal1 and NCAPG expression were associated with OS in GC patients (all *P* < 0.001, Table 2). Multivariate Cox regression analysis indicated that tumor diameter, TNM stage, Gal1 and NCAPG expression were independent prognostic factors for GC (Gal1: HR, 0.072, 95% CI, 0.035–0.145, *P* < 0.001; NCAPG: HR, 0.185, 95% CI, 0.081–0.420, *P* < 0.001, Table 3).

**Table 2:** Univariate Cox regression analysis of clinicopathological variables, Gal1 and NCAPG in patients with GC.

**Variable**	***n* = 145**	
	**HR (95% CI)**	***P***
Sex (male vs. female)	0.909 (0.580–1.423)	0.675
Age (≤65 years vs. >65 years)	1.211 (0.788–1.860)	0.382
Tumor diameter (≤5 cm vs. >5 cm)	3.117 (2.022–4.805)	<0.001
Pathological classification (I/II vs. III)	1.810 (0.789–4.153)	<0.001
Depth of invasion (T2/T3 vs. T4)	5.447 (2.203–13.473)	<0.001
Lymph node metastasis (N0 vs. N1*–*N3)	1.454 (1.221–1.731)	<0.001
TNM stage (I*–*II vs. III)	0.184 (0.074–0.454)	<0.001
Gal1 state (low vs. high)	12.825 (6.704–24.534)	<0.001
NCAPG state (low vs. high)	6.325 (2.908–13.757)	<0.001

**Table 3:** Multivariate Cox regression analysis of clinicopathological variables, Gal1 and NCAPG expression in patients with GC.

**Variable**	**B**	***n* = 145**		
		**Wald**	**HR (95% CI)**	***P***
Tumor diameter (≤5 cm vs. >5 cm)	−0.691	8.579	0.501 (0.316–0.795)	0.003
TNM stage (I–II vs. III)	−2.670	6.868	0.069 (0.009–0.510)	0.009
Gal1 state (low vs. high)	−2.632	53.153	0.072 (0.035–0.145)	<0.001
NCAPG state (low vs. high)	−1.689	16.235	0.185 (0.081–0.420)	<0.001

### Synergistic effect of Gal1 and NCAPG on OS and DFS

The patients were stratified into three groups based on the IRS for Gal1 and NCAPG, i.e., both high (*n* = 73), either Gal1 or NCAPG high (*n* = 47), and both low (*n* = 25). Kaplan-Meier analysis revealed that the both low expression group had more favorable OS and DFS, while the both high expression group had poorer OS and DFS, compared to the Gal1 or NCAPG high group (*P* < 0.001, Figure 2E, 2H).

### Gal1 regulates NCAPG at mRNA and protein levels

**Figure 3:**
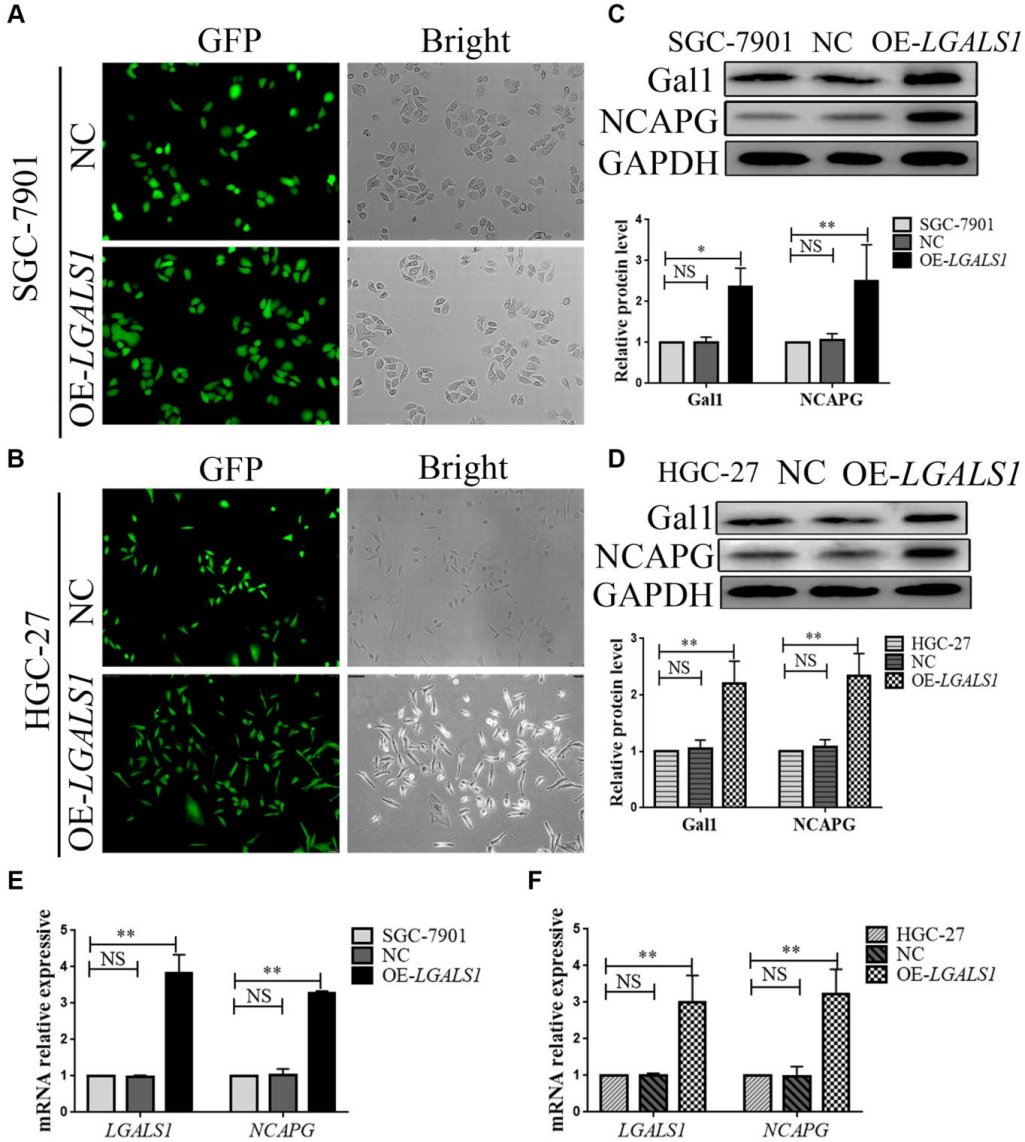
***LGALS1* regulates *NCAPG* at the mRNA and protein levels.** Transduction efficiency confirmed by green fluorescent protein (GFP) assay in (**A**) SGC-7901 cells and (**B**) HGC-27 cells. Original magnification 200×. (**C**, **D**) Western blot confirmation of stable overexpression of Gal1 and NCAPG in (**C**) SGC-7901 and (**D**) HGC-27 cells when *LGALS1* was overexpressed. (**E**, **F**) Quantitative real-time PCR (qRT-PCR) analysis of *LGALS1* and *NCAPG* expression in (**E**) SGC-7901 and (**F**) HGC-27 cells when *LGALS1* was overexpressed. Abbreviations: OE-*LGALS1*: Overexpression of *LGALS1*; NC: negative control (empty vector); NS: not significant. ^*^P < 0.05; ^**^P < 0.01.

We constructed lentivirus that overexpressed *LGALS1* in SGC-7901 and HGC-27 cells to obtain four cell lines, overexpressed LV-*LGALS1* SGC-7901 (OE-*LGALS1* SGC-7901), overexpressed LV-*LGALS1* HGC-27 (OE-*LGALS1* HGC-27), and corresponding transfected negative control (NC) cells. Successful overexpression of *LGALS1* was confirmed by green fluorescent protein signal (Figure 3A, 3B), Western blotting (Figure 3C, 3D), and qRT-PCR (Figure 3E, 3F). Western blotting indicated that OE-*LGALS1* SGC-7901 and OE-*LGALS1* HGC-27 cells had higher Gal1 levels than NC cells and wild cells (*P* < 0.05 and < 0.01, respectively, Figure 3C, 3D). mRNA expression for *LGALS1* was detected using qRT-PCR; the expression of *LGALS1* mRNA was consistent with Gal1 protein expression (all *P* < 0.01, Figure 3E, 3F).

In all cell lines, NCAPG expression was consistent with Gal1 expression on Western blotting, and was significantly higher in OE-*LGALS1* GC cells than in NC cells and wild cells (all *P* < 0.01, Figure 3C, 3D). We then performed qRT-PCR to evaluate *NCAPG* mRNA expression in all cell lines, and the results were consistent with those of Western blotting (all *P* < 0.01, Figure 3E, 3F). This indicated that *LGALS1* could positively regulate *NCAPG* expression at mRNA and protein levels.

### *LGALS1* and *NCAPG* mutually reinforced GC cell line regulation

**Figure 4:**
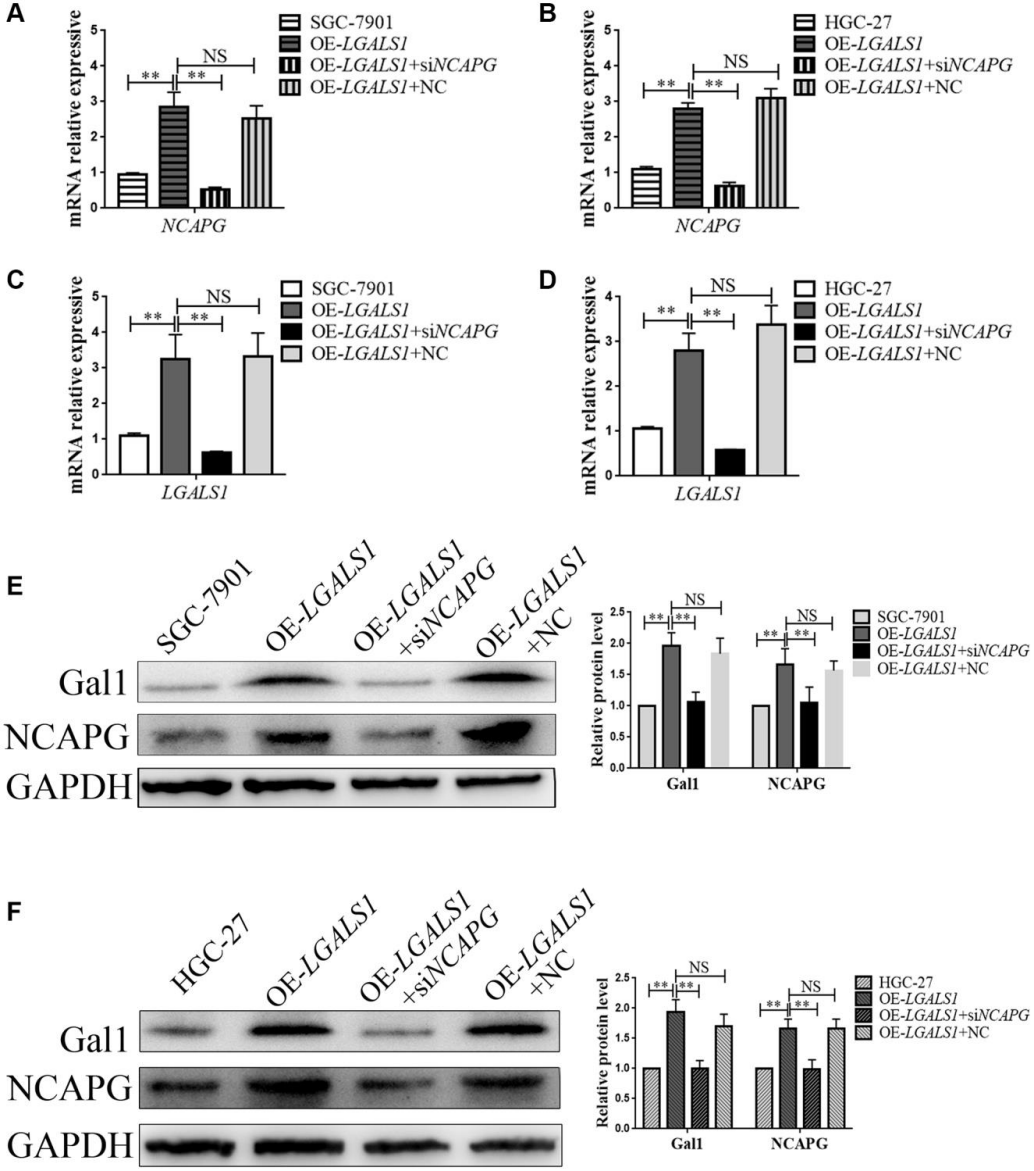
***LGALS1* and *NCAPG* may mutually reinforce regulation in GC cell lines.** qRT-PCR analysis of (**A**, **B**) *NCAPG* and (**C**, **D**) *LGALS1* expression in SGC-7901 and HGC-27 cells when *LGALS1* was overexpressed, with or without simultaneous silencing of *NCAPG*. Western blot confirmation of stable overexpression of Gal1 and NCAPG in (**E**) SGC-7901 and (**F**) HGC-27 cells when *LGALS1* was overexpressed, with or without simultaneous silencing of *NCAPG*. Abbreviations: OE-*LGALS1*: Overexpression of *LGALS1*; si*NCAPG*: silencing of *NCAPG*; OE-*LGALS1*+NC: overexpression of *LGALS1* + *NCAPG* negative control (empty vector); NS: not significant. ^*^P < 0.05; ^**^P < 0.01.

In order to further explore the regulatory relationship between *LGALS1* and *NCAPG*, lentiviral was used to generate overexpressed LV-*LGALS1* SGC-7901 and HGC-27 stable cell lines. We constructed an siRNA to simultaneous silence *NCAPG* expression in these stable cell lines, and obtained OE-*LGALS1*+ *NCAPG*-knockdown GC cells (OE-*LGALS1*+si*NCAPG*), including both SGC-7901 and HGC-27 cell lines. mRNA expressions for *LGALS1* and *NCAPG* were detected using qRT-PCR. *NCAPG* knockdown in OE-*LGALS1* GC cell lines significantly inhibited *NCAPG* mRNA expression for SGC-7901 (*P* < 0.01, Figure 4A) and HGC-27 (*P* < 0.01, Figure 4B). However, *LGALS1* mRNA expression was also decreased when *NCAPG* was knocked down in OE-*LGALS1* GC cell lines, including SGC-7901 (*P* < 0.01, Figure 4C) and HGC-27 (*P* < 0.01, Figure 4D). Thus, *LGALS1* and *NCAPG* mutually reinforced GC cell line regulation. In order to further verify these results, Western blotting was used to detect Gal1 and NCAPG protein expressions in each GC cell line, and the results were consistent with those of qRT-PCR (all *P* < 0.01, Figure 4E, 4F).

### *LGALS1* promoted migration and invasion in GC cells through *NCAPG* regulation

**Figure 5:**
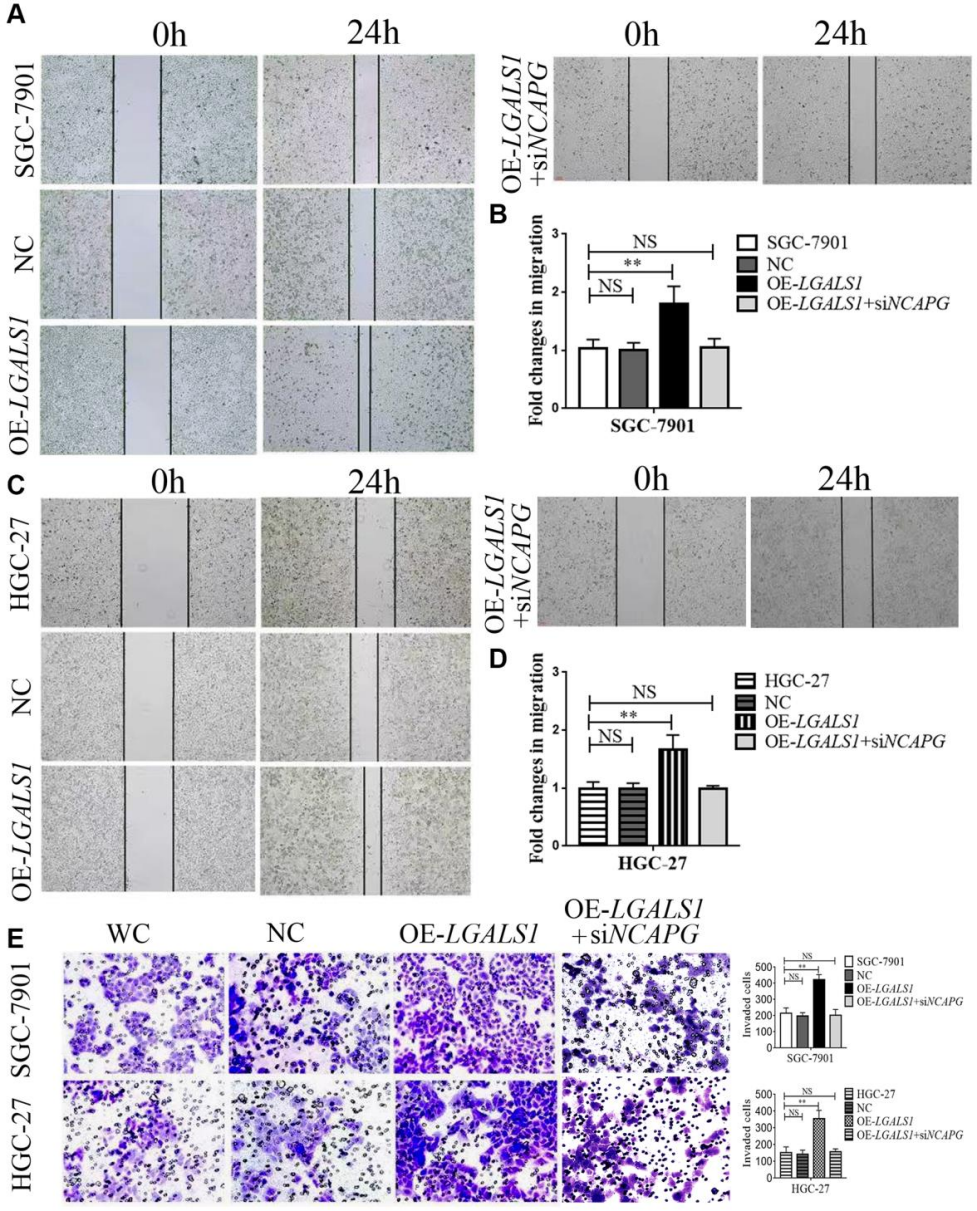
***LGALS1* promotes the migration and invasion of GC cell lines *in vitro*.** Overexpression of *LGALS1* (OE-*LGALS1*) significantly enhanced the migration capacity of (**A**, **B**) SGC-7901 and (**C**, **D**) HGC-27 cells compared with wild control (WC) and negative controls (NC). The migration capacity was abolished when *NCAPG* was simultaneously silenced. Magnification: ×100. (**E**) Transwell assay showing that overexpressed *LGALS1* significantly enhanced the invasion ability of SGC-7901 and HGC-27 cells, and simultaneous silencing of *NCAPG* abolished the invasion capacity (*n* = 3). Magnification: ×200. Abbreviations: NC: negative control (empty vector); OE-LGALS1: Overexpression *LGALS1*; OE-LGALS1+siNCAPG: Overexpression of *LGALS1* +silencing of *NCAPG*. NS: not significant. ^**^P < 0.01.

Wound healing and transwell assays were used to assess migration and invasion abilities of SGC-7901 and HGC-27 cells, and compare them to the NC (OE-*LGALS1* transfected NCs), OE-*LGALS1*, and OE-*LGALS1*+si*NCAPG* groups. The wound healing experiment demonstrated that *LGALS1* overexpression enhanced SGC-7901 migration. However, this was partly rescued by reinfected *NCAPG*-siRNA in OE-*LGALS1* GC cells (Figure 5A). Figure 5B shows the fold changes in migration. These experiments were repeated in HGC-27 cells to confirm *LGALS1*-mediated promotion of migration *in vitro*, which was reduced by simultaneous *NCAPG*-knockdown (Figure 5C, 5D).

Transwell assay showed significantly enhanced invasion ability in OE-*LGALS1*, SGC-7901, and HGC-27 cells. The cell invasion capability could be partly rescued by re-infected *NCAPG*-siRNA in OE-*LGALS1* GC cells (Figure 5E; *P* < 0.01).

## DISCUSSION

GC has a very high mortality rate, with over a million new cases and 769,000 estimated deaths reported in 2020 [1]. In China, approximately 80% of the patients diagnosed with GC have advanced metastatic disease [20]. This is because of the lack of non-invasive examination methods and sensitive screening markers. Although the long-term survival rate for GC patients has improved in China since 2000 [21], it still remains unsatisfactory [22]. Recurrence and metastasis after surgery are the main causes of death in advanced GC. Although TNM staging is the gold standard for prognosis assessment of GC patients, it cannot accurately predict the risk of postoperative recurrence and metastasis. Therefore, there is an urgent need to develop novel methods for diagnosis, treatment, and prognosis evaluation for GC patients, and to provide therapeutic targets for targeted therapy.

Previous studies have found that Gal1 promotes cell shedding, homotype cell aggregation, migration, invasion, adhesion, and angiogenesis in tumors [13]. Previous studies have also demonstrated that high Gal1 expression in GC-associated fibroblasts induced epithelial-mesenchymal transition and enhanced GC cell invasion and metastasis [23].

Galactolectins play a variety of roles in normal physiology. In cancer, they are often expressed at elevated levels and associated with poor prognosis [24]. They are known to contribute to various cancer progression pathways by interacting with cancers and matrix glycans [24]. Research on the regulation of biological behavior of GC by galactolectins has focused on Gal1, Gal-3, and Gal-9 [24–26]. Previous studies have shown that Gal1 expression in GCTs is associated with poor prognosis in GC patients [27, 28]. The present study showed that Gal1 was significantly higher in GCTs than in the adjacent non-cancerous tissues, and that its expression was correlated with tumor diameter, pathological classification, depth of invasion, lymph node metastasis, and TNM stage. All these pathological parameters are also correlated with GC prognosis. Kaplan-Meier survival analysis showed that high Gal1 expression in GCTs was significantly correlated with postoperative OS and DFS in GC patients. These results suggest that Gal1 expression may regulate the malignant biological behavior of GCTs and is closely related to patient prognosis. Therefore, Gal1 may be used as an indicator for the prognosis of GC patients. However, the mechanism by which Gal1 affects GC prognosis has not been fully elucidated.

In this study, we found that Gal1 expression in GCTs was closely related to NCAPG expression, which has not been reported in previous studies. NCAPG is a subunit of the agglutinate protein complex that is responsible for chromosomal cohesion and stability during cell division [29]. According to gene ontology analyses, NCAPG-related pathways include the cell cycle, mitosis, and cell-cycle chromosome premetaphase coagulation. Previously, RNA-seq was performed on tissue samples from 95 human individuals, representing 27 different tissues, in order to determine the tissue specificity of the protein-coding genes [30]. NCAPG is mainly expressed in the bone marrow, lymph nodes, and testes in healthy individuals. Several recent studies have found abnormal NCAPG expression in a variety of tumors, including GC, which affects their prognosis [14, 15, 17, 18, 31]. It has been demonstrated that NCAPG expression in GCTs can predict the prognosis of GC patients [19]. However, the expression and functional role of NCAPG in GC remain unclear. We observed that NCAPG expression in GCTs was higher than in the adjacent tissues. NCAPG expression in GCTs was also positively correlated with gender, tumor diameter, pathological classification, depth of invasion, lymph node metastasis, and TNM stage. Although the association between gender and GC prognosis has not been reported, all the remaining factors are known prognostic indicators in GC patients. Furthermore, several gender-dependent diagnostic markers for GC have been reported, including Kindlin-1, an adhesion protein member of the integrin-interacting proteins [32]. Therefore, the relationship between NCAPG expression and gender in GC requires further analysis.

Kaplan-Meier survival analysis also showed that high Gal1 expression in GCTs was significantly correlated with postoperative OS and DFS, which was consistent with previous studies. Gal1 and NCAPG were also found to have a synergistic effect. Cox regression model confirmed that high Gal1 and NCAPG expressions were effective independent prognostic factors in all patient groups.

In order to explore the relationship between Gal1 and NCAPG, we conducted an *in vitro* experiment. We found that Gal1 regulated NCAPG expression at mRNA and protein levels. When we knocked down *NCAPG* in OE-*LGALS1* GC cells, Gal1 expression also decreased. This indicates that Gal1 and NCAPG may regulate each other. Existing literature suggests that *NCAPG* promotes oncogenesis in non-small cell lung cancer cells by upregulating *LGALS1* expression [14]. Our study was the first to demonstrate that *LGALS1* regulates *NCAPG* expression in GC, and that *LGALS1* and *NCAPG* may be mutually regulated. Further *in vitro* analysis confirmed that *LGALS1* promoted GC invasion and metastasis through *NCAPG* regulation.

The regulation of GC invasion and migration by NCAPG has also been reported previously [33]. NCAPG overexpression in GC cell lines decreased the levels of caspase-3, Bax, and E-cadherin, but elevated Bcl-2, vimentin, N-cadherin, Snail, and Slug levels. NCAPG overexpression also increased the expression of Wnt1, phosphorylated GSK3beta, and total beta-catenin, while decreasing the expression of phosphorylated catenin. Functionally, NCAPG overexpression improved the anti-apoptotic ability of GC cells and promoted their Epithelial-mesenchymal Transition (EMT), making them more aggressive and mobile. In conclusion, NCAPG overexpression may promote EMT and inhibit tumor cell apoptosis by activating the Wnt/β-catenin signaling pathway [33]. Several genes can promote GC metastasis by activating the Wnt/β-catenin signaling pathway [34, 35]. In addition, Wnt/β-catenin signaling pathway activation can also promote GC proliferation, apoptosis [36], and ferroptosis resistance [37]. However, the mechanism by which *LGALS1* regulates *NCAPG* has not been reported. Subsequent studies may explore these molecular mechanisms in GC cells through single-cell sequencing and other experiments to develop new therapeutic targets.

In summary, Gal1 and NCAPG could be prognostic molecular biomarkers for GC. Gal1 promotes GC cell invasion and migration through targeted regulation of NCAPG *in vitro*. Notably, the combination of Gal1 and NCAPG was an efficient prognostic indicator for GC and their synergistic effect is reported for the first time. Further investigations of the role of these proteins may provide new opportunities for novel GC treatment strategies.
